# Psychotherapists’ Reports regarding the Impact of the COVID-19 Pandemic on Their Patients: A Cross-National Descriptive Study Based on the Social-Ecological Model (SEM)

**DOI:** 10.3390/ijerph19116825

**Published:** 2022-06-02

**Authors:** Yvonne Schaffler, Martin Kuska, Antonia Barke, Bettina K. Doering, Katharina Gossmann, Zdenek Meier, Natalia Kascakova, Peter Tavel, Elke Humer, Christoph Pieh, Peter Stippl, Wolfgang Schimböck, Barbara Haid, Thomas Probst

**Affiliations:** 1 Department for Psychosomatic Medicine and Psychotherapy, Danube University Krems, 3500 Krems, Austria; martin.kuska@donau-uni.ac.at (M.K.); elke.humer@donau-uni.ac.at (E.H.); christoph.pieh@donau-uni.ac.at (C.P.); thomas.probst@donau-uni.ac.at (T.P.); 2 College of Applied Psychology, 41155 Terezin, Czech Republic; 3 Clinical and Biological Psychology, Catholic University of Eichstätt-Ingolstadt, 85072 Eichstätt, Germany; antonia.barke@ku.de (A.B.); katharina.gossmann@ku.de (K.G.); 4 Clinical Psychology and Psychotherapy, Brandenburg Medical School Theodor Fontane, 16861 Neuruppin, Germany; bettina.doering@mhb-fontane.de; 5 Olomouc University Social Health Institute (OUSHI), Palacky University Olomouc, 77111 Olomouc, Czech Republic; zdenek.meier@oushi.upol.cz (Z.M.); natalia.kascakova@oushi.upol.cz (N.K.); peter.tavel@oushi.upol.cz (P.T.); 6 Psychiatric-Psychotherapeutic Outpatient Clinic, Pro Mente Sana, 81108 Bratislava, Slovakia; 7 Austrian Federal Association for Psychotherapy, 1030 Vienna, Austria; oebvp.stippl@psychotherapie.at (P.S.); wolfgang.schimboeck@liwest.at (W.S.); haid@transformberatung.com (B.H.)

**Keywords:** psychotherapy, COVID-19, pandemic, stressors, social environment, mental health, adaptive responses, maladaptive responses

## Abstract

The COVID-19 outbreak has raised questions about how vulnerable groups experience the pandemic. Research that focuses on the view of individuals with pre-existing mental health conditions is still limited, and so are cross-country comparative surveys. We gathered our sample of qualitative data during the first lockdown after governmental measures against the spread of the SARS-CoV-2 virus came into force in Austria, Czechia, Germany, and Slovakia. A total of *n* = 1690 psychotherapists from four middle European countries answered the question of how the COVID-19 pandemic was addressed in sessions by their patients during the early stage of unprecedented public health conditions. We employed a descriptive qualitative methodology to determine themes following levels of the social-ecological model (SEM) regarding how the COVID-19 pandemic affected patients. At the public policy level, stressful environmental conditions concerned the governmental mitigation efforts. At the level of community/society, reported key themes were employment, restricted access to educational and health facilities, socioeconomic consequences, and the pandemic itself. Key themes at the interpersonal level regarded forced proximity, the possibility of infection of loved ones, childcare, and homeschooling. Key themes at the individual level were the possibility of contracting COVID-19, having to stay at home/isolation, and a changing environment. Within the SEM framework, adaptive and maladaptive responses to these stressors were reported, with more similarities than differences between the countries. A quantification of word stems showed that the maladaptive reactions predominated.

## 1. Introduction

In the first half of 2020, people worldwide found themselves in an unprecedented situation, as COVID-19 and lockdowns elicited the progressive emergence of several types of psychological distress [1,2,3,4]. A critical cause of psychological distress is reduced social interaction due to governmentally imposed measures to curb the spread of the virus, known as “social distancing” [5,6]. This measure elevates the risk for social isolation and loneliness [7], leading to a substantial level of distress, including frustration, infection fears, misperception of the danger of the situation, and post-traumatic stress symptoms [8,9]. From the socio-ecological perspective, infectious diseases yield concerns at multiple levels, from the public policy level to the community/society, to the interpersonal, to the individual, depending on a given environment (such as a country whose government decides over the measures taken) and timeframe (as the course of a pandemic with its concomitant mitigation efforts changes over time) [10,11]. These concerns are causing multidimensional and interconnected psychological, social, and ecological effects that influence people’s behavioural [12] and emotional [13] responses. Emotional responses are likely to include fear and panic [14], also known to have occurred during previous infectious outbreaks [15]. As described by novelist Jack London at the beginning of the 20th century, scarlet fever brought drinking, robbing, and sometimes even killing, and more adaptive behaviours such as fleeing and self-isolating [16]. Immediate reactions to COVID-19 likewise have been documented to cover a wide range, including panic buying and hoarding behaviour [17] and stigmatisation of ill people or vulnerable groups [18,19]. On the more adaptive end, individuals were found to have followed the preventive measures advocated by the WHO, observed hygiene recommendations [20], and tried to maintain their daily routine [21].

Not only do the responses to complex disasters vary from person to person, as some experience the pandemic as a heavy burden, while others adapt well to the situation [21], but vulnerability pathways affect populations in different ways and to a varying degree [22]. Particularly younger adults, women, people without work, and those with low income were vulnerable to a stressful experience of the COVID-19 pandemic and concomitant measures [23]. Vulnerable populations also include individuals with special health care needs, particularly those with chronic health conditions, including mental health conditions [22,24]. Most existing studies suggest that individuals with pre-existent mental health conditions also had worse mental health outcomes during the first lockdown phase [25,26]. However, studies on individuals with mental health problems do not generally suggest worse outcomes during the first lockdown phase [27,28,29]. Moreover, although people with enhanced levels of depression, anxiety, and stress symptoms are prone to maladaptive responses to disaster [30], depression and anxiety symptoms did not significantly predict the burden experienced through the disaster [21].

Qualitative research exploring the experiences and perceptions of how life has changed at this time is scarce but needed, as it could provide clarity on the actual impact of the pandemic on people with pre-existing mental health conditions [31]. Common factors that have negatively impacted the health outcomes of individuals with pre-existing mental health conditions are a rapidly changing environment, risk of infection, increasing isolation, and reduced access to support services [25,32]. Looking into the challenges faced by people with mental health conditions, a study relying on a UK adult sample of 22 patients found the following five factors that were subjectively contributing to a deterioration in their mental health: “feeling safe but isolated at home,” “disruption to mental health services,” “cancelled plans and changed routines,” “uncertainty and lack of control”, and “rolling media coverage” [33]. Investigating the personal experiences of people with anxiety, depression, and obsessive-compulsive disorder during COVID-19 by analysing 130 posts in subreddit forums, Brewer et al. [31] found reports on the “intensifying of symptoms” and a “lack of social support” to be the most common crosswise themes for all forum types. A qualitative study from the UK drawing on a large, ethnically diverse sample found that many participants’ existing mental health difficulties were exacerbated. In addition, they experienced specific psychological impacts of the pandemic, struggles with social connectedness, and inadequate access to mental health services. At the same time, some found new ways to cope and connect to the community [34].

Since previous findings suggest a complex interaction of factors, with a strong focus on the psychological impact of the socio-economic and political context [35], researchers have proposed the social-ecological model (SEM) to organise the stressors and reactions of specific vulnerable populations [36]. A SEM [19,37] can provide a visual image of the interplay of public policy, community/society-, interpersonal-, and individual-level factors that can lead to increased risks of infection and associated morbidity and mortality for individuals and groups. Moreover, it can highlight people’s response to environmental challenges, commonly described as risk factors, such as the ecological changes brought about by a pandemic and subsequent public health measures to curb viral transmission. It helps to explore how the structural properties of a specific environment can produce different responses, ranging from more maladaptive to more adaptive at a particular point in time. In this vein, Moore et al. [13] accessed an adult sample of sufferers of anxiety and depression from Arkansas through an online instrument during July and August of 2020 to examine how COVID-19 affected anxiety and depression symptoms through the social-ecological lens. They found the following emergent themes at the individual level: “isolation/loneliness,” “fear of contracting COVID-19”, and “uncertainty about the future.” Themes at the interpersonal level were: “fears of family contracting COVID-19”, “separation from family members,” and “domestic relationships.” Themes at the level of community and societal stressors were: “employment,” “community and societal systems,” “media,” and the “COVID-19 pandemic”. We aim at a similar endeavour focusing on several countries from Europe and emphasising reactions that are either adaptive or maladaptive for the maintenance of an individual’s health in the situation of viral spread.

The pandemic has placed people in states of existential threat and limited freedom, eliciting responses on various levels, from public policy to the individual level. No prior qualitative studies have explored the variety of immediate reactions to COVID-19 of people with pre-existing mental illness across nations. The SEM has proven to be a valuable tool to systematically guide research in specific settings and at one particular time.

In this study, we focus on the reports of Austrian, Czech, German, and Slovak psychotherapists about their patients’ experiences of the COVID-19 pandemic during the first lockdown that was implemented to prevent the spread of the disease. To capture the variability of patients’ experiences, we selected psychotherapists who are experts in assessing their patients’ reactions and thus may also capture reactions that patients cannot verbalise. We also approached patients indirectly so as not to place additional stress on them during this challenging phase. This study thus aims to look at how COVID-19 was addressed during psychotherapy sessions by mental health patients in treatment, how the addressed themes fit within the conceptual framework of the SEM, and which themes are specific to a particular geographical area and in what way.

## 2. Materials and Methods

### 2.1. Study Design, Samples, and Data Collection

We conducted a cross-sectional online survey, drawing on a descriptive approach to qualitative research [38,39]. Qualitative descriptive designs are common in healthcare research. They are used in areas where little is known about the topic under investigation and in studies that aim to stay close to and describe participants’ experiences [40].

Qualitative and descriptive data were collected through Research Electronic Data Capture (REDCap, Nashville, TN, USA), a widely used web-based software to capture study responses and participant consent [41]. In contrast to previous qualitative studies that drew upon data from rather small samples from one country, a research registry, or internet forums, we generated original data from four different countries. Our study was part of a larger study examining the provision of psychotherapy at the beginning of the COVID-19 pandemic among Austrian [42], Czech, German, and Slovak psychotherapists [43]. In total, 1885 psychotherapists participated (A: 1547, CZ: 112, DE: 130, SK: 96). In Austria, participants averaged 51.67 (standard deviation (SD) = 9.69) years old, and 75.7% were female (compared to 74.1% female in the Austrian list of psychotherapists in March 2020) [42].

In the other countries, the mean age of participants was 46.70 (SD = 10.68) years, with 77.8% of the psychotherapists being female [43]. Of the total number of participants, 1690 gave answers in short written form to the open question: “How do your patients address COVID-19 in the psychotherapeutic sessions?”. We assumed that the interviewed psychotherapists would answer this question by either stating what topics their patients brought in or how they brought in their concerns if they did so in a non-linguistic manner. The length of psychotherapists’ written statements ranged from using only one word to a maximum of eight words. Some answered in a more comprehensive way using complete sentences. It is vital to note that patient numbers per participating therapist varied according to the therapists’ work situation, ranging between one and 25 patients per week, with an average of M = 10.12 (SD = 9.05) patients per week in Austria [42], M = 14.37 (SD = 11.44) in Czechia, M = 24.38 (SD = 12.65) in Germany, and M = 14.71 (SD = 11.60) in Slovakia [43].

The psychotherapists who participated in our study agreed to the privacy statement to start the survey (informed consent). Participation was voluntary, without incentives. The principles of the Declaration of Helsinki were adhered to, and the Ethics Committee approved the study of the Danube University Krems, Austria.

### 2.2. Sample Recruiting

In Austria, all psychotherapists registered on the official Austrian psychotherapist list were invited by e-mail (approximately *n* = 6000 with valid e-mail addresses).

In Czechia, the psychotherapists were contacted through the e-mail list of the Czech Association for Psychotherapy (https://czap.cz/, accessed on 1 May 2020), a Czech national association joining a high number of Czech psychotherapists.

In Germany, all e-mail addresses were gathered from the publicly available directories of four different regional and national psychotherapeutic associations. In these directories, the associations publish the contact information of all licensed psychotherapists who gave their consent to such publication.

In Slovakia, e-mails with information about an online survey were sent to the chairman of the Slovak Psychotherapeutic Society and then to the chairpersons of particular psychotherapeutic societies and then sent from these sources to psychotherapists via e-mail lists. The psychotherapists who were interested in participating filled in an online questionnaire. The data were then automatically sent to a central data set.

### 2.3. Data Gathering Period

**Figure 1:**
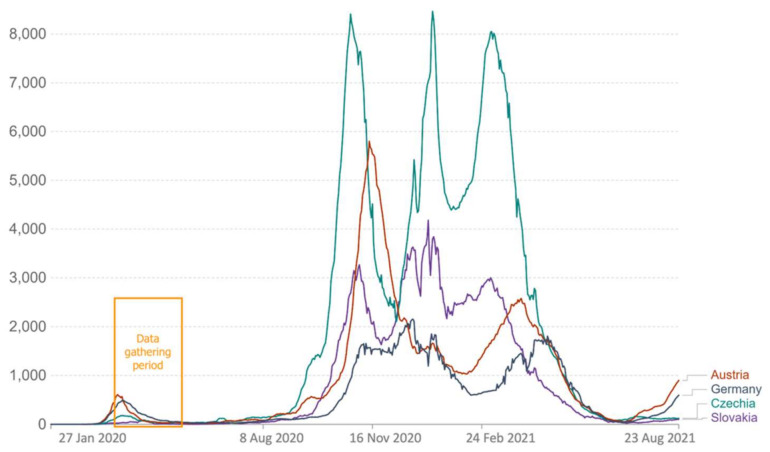
The weekly cases per million people in all four countries within the data gathering period. Source: Johns Hopkins University CSSE COVID-19 Data.

**Figure 2:**
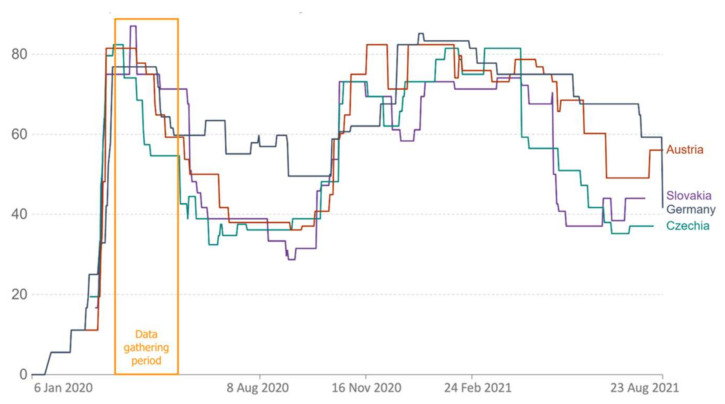
The strictness of the mitigation efforts in all four countries within the data gathering period. The composite measure is based on nine response indicators including school closures, workplace closures, and travel bans, rescaled to a value from 0 to 100 (100 = strictest). If policies vary at the subnational level, the index is shown as the response level of the strictest sub-region. Source: Hale, T.; Angrist, N.; Goldszmidt, R.; et al. A global panel database of pandemic policies (Oxford COVID-19 Government Response Tracker). *Nat. Hum. Behav.* **2021**, *5*, 529–538. https://doi.org/10.1038/s41562-021-01079-8.

In Austria, the survey was open from 24 March to 1 April 2020; in Czechia, the survey was open from 6 May 2020, until 20 May 2020; in Germany, from 19 May 2020, until; and in Slovakia, from 8 May 2020, until 22 May 2020. For Austria, this was about eight days after lockdown measures were initiated. The survey in Austria thus took place in the initial phase of the lockdown in Austria when no restrictions were lifted yet. For Czechia, this was about seven weeks after lockdown measures were initiated and about two weeks after restrictions began to be lifted; however, most stay-at-home regulations were still in place. For Germany, this was about eight weeks after lockdown measures were initiated and about two weeks after restrictions began to be lifted; however, most stay-at-home regulations were still in place. For Slovakia, this occurred seven weeks after lockdown measures were initiated and about three weeks after restrictions began to be lifted; however, most stay-at-home regulations, including closed schools and kindergartens, were still in place. We chose the earliest possible point in time to collect data after lockdowns began. Since international data gathering relied on cooperation, not all data could be collected simultaneously. Figure 1 and Figure 2 show very similar epidemic conditions regarding weekly confirmed cases (Figure 1) and strict mitigation efforts (Figure 2) in all four countries when collecting data.

### 2.4. Analysis

We used an approach to content analysis that combined deductive with inductive coding [44] to depict the range of topics we could find regardless of their frequency. First-order categories were theory-based and directly derived from the SEM. They comprise an individual, an interpersonal, a combined community/societal and a public policy level. We then, also deductively, divided the first-order categories into three clusters, namely into (a) environmental conditions or themes that directly refer to an addressed environmental disruption, (b) responses that we considered maladaptive, as they could potentially lead to a deterioration of an individual’s health state, and (c) adaptive responses that we interpreted as more conducive to an individual’s health. For example, to prevent infection with COVID-19, people should avoid meeting potentially infectious family members, friends or colleagues. Adaptive responses, therefore, involve compliance with the mitigation efforts. On the other hand, non-compliance with the measures may entail infection and is therefore considered maladaptive. Also considered maladaptive is the experience of psychological stress [45,46], the perception of isolation [47] or insomnia [48] because such responses may weaken the immune function, entail a deterioration of psychological health, and lead to imprudent actions in an epidemic situation.

Since the levels of the SEM are intertwined, we assigned some themes to more than one level. For example, we consider schooling an institutional and thus communal/societal issue, but homeschooling also has interpersonal aspects. Therefore, we mentioned home learning aspects (such as distress because of additional teaching) at the level of community/society, but interpersonal aspects of homeschooling (such as the burden of childcare) at the interpersonal level.

Within each cluster, we clustered lower-order categories inductively. Since we did not explicitly ask how COVID-19 has affected the patient’s mental health, we received answers that either included a connection between a response and a stressor (such as “worries about economic deterioration”) or answers that only referred to a response without pointing to a stressor (such as “loneliness”). We left the connection intact if the response and stressor were semantically connected (such as “worries about economic deterioration”). We abstained from quantifying our qualitative responses, as our regional samples, except the Austrian one, were not large enough for such an endeavour. Thus, it is essential to note that some reactions were only mentioned once. Therefore, our qualitative results should be understood as a texture of responses that provide an image of each country’s patients’ concerns.

We triangulated the qualitative data with descriptive statistics of the five most frequent word stems in the psychotherapists’ responses from each country to give a numerical impression of the most frequent responses. To this end, the entire text corpus was analysed to identify the most frequently used word stems using the word stem analysis method [49,50]. A total of 4293 word stems were identified and manually checked to avoid possible different semantic meanings of the word stems in the individual testimonies. This task was performed separately for three languages. Finally, English words representing each word stem in German, Czech, and Slovak were linked (see Table 1 for details).

We used ATLAS.ti vers. 8, a qualitative data analysis tool for coding and counting.

### 2.5. Ethics

This study was conducted in accordance with the Declaration of Helsinki and approved by the Ethics Committee of Danube University Krems, Austria (ethical number: EK GZ 23/2018–2021). All participants gave electronic informed consent to participate and complete the questionnaires. Data were collected anonymously without IP addresses or GPS tracking, and the data protection officer approved this procedure of Danube University Krems.

## 3. Results

First, we narratively report themes according to the levels of our modified version of the SEM. Second, we visualised themes for the addressed stressful environmental conditions and maladaptive and adaptive responses. Third, we report the most frequent word stems used in the testimonies according to each country (Tables S1–S4).

### 3.1. Narrative Reports of Themes

The narrative reports address the themes derived from the four levels of our modified version of the SEM, i.e., the individual level, the interpersonal level, a combined community and societal level, and a level of public policy, each of which is divided into addressed stressful environmental conditions, maladaptive responses, and adaptive responses.

#### 3.1.1. Public Policy

Addressed stressful environmental conditions at the public policy level found in all four countries regarded the governmental efforts to mitigate the viral spread. According to the SEM, maladaptive responses included fears revolving around government actions, particularly the possibility of a complete curfew or a permanent change of rules and regime. Fears were also directed at police sanctions, as patients would “not dare go outside” or experienced a “loss of personal freedoms due to governmental influence.” Patients also expressed fears regarding authorities and government entities’ perceived might or even a comeback of totalitarianism. Anger and resistance were reported as directed towards the governmentally imposed restrictions. One therapist explained the observed intrapsychic dynamics: “Patients always question the measures’ effectiveness. They cite examples such as that they see police or politicians crowded together in the streets without wearing face masks. They ask why the authorities do not comply with the measures but impose fines on citizens. This discrepancy triggers anger and frustration among patients. Eventually, they resign and comply with the regulations for fear of punishment, which triggers strong reluctance. Patients describe sleep disturbance and irritable moods due to these inner conflicts”.

Further reported reactions towards the mitigation efforts were impatience as to how long the restrictions would last, the inability to deal with measures, the inability to see family members who remained abroad because they did not want to submit to the state quarantine regulations, and the experience of being overruled by public policy as “no one has thought about my particular situation”. Further addressed was a reluctance to wear a mask as wearing a mask would cause discomfort or lead to anxious states, including the experience of lack of oxygen and thus a general concern about coping with being outside. In contrast, psychotherapists also observed that patients would overidentify with the measures because they could not understand how anyone could transgress the hygiene measures.

Regarding more adaptive responses, we found that mitigation efforts were accepted and respected, factually discussed, and seen from the perspective that “the state takes care of me so that I remain healthy”.

#### 3.1.2. Community and Societal Level

Patients in all four countries addressed the following stressful environmental conditions: Changed conditions at (or loss of) work, restricted access to educational and health facilities, socioeconomic consequences, and the pandemic itself.

The addressed maladaptive responses referred to difficulties adjusting to working from home, which entailed distress due to excessive workloads and conflicts with colleagues. Other work-related problems referred to a loss of employment, which inspired feelings of helplessness, worry, and future anxiety or to a limited working ability, which would lead to financial hardship and not being able to maintain one’s family. As schools were closed in all four countries, educational themes were related to high levels of distress caused by having to teach at home, the uncertainty of learning progress, worry about the quality of education, feeling left alone by the school and difficulties in motivating children to learn from home. At the time of the survey in Germany, children were about to resume classes, which sparked worries that they would become infected. The restricted access to medical facilities, which included the closure of psychiatric outpatient services, contributed to a loss of daily structure and an aggravation of symptoms, particularly in severe mental illness or trauma. The non-availability of the usual medical infrastructure would spark fear in patients, for example, to give birth under the given circumstances. A further cause for concern was assumed loneliness among individuals in hospitals and nursing homes. Themes regarding the psychotherapeutic setting included concerns that personal contact would remain interrupted. Some patients found remote therapy challenging to sustain because they had no private space available at home. Another addressed problem was that they feared they would violate the exit restrictions on their way to see their psychotherapist.

Media reports were mentioned to induce fear, notably as images from Italy or Spain showed rows of coffins to illustrate the high numbers of deaths due to COVID-19. The increased media consumption would lead to distress and supersaturation with COVID-19 related media reports, which sometimes entailed patients trying to avoid viewing the news. Addressed media-related subthemes regarded difficulties in finding the necessary information and consuming fake news. Conspiracy theories about the pandemic’s origins and assumed “societal cleansing” effects were reported to have emerged. Other reactions were the denial or trivialising of the pandemic. One patient was reported to have said: “I would gladly trade place with those infected by corona; an infection is a matter of a few weeks, but my problem has been bothering me for years...”. The pandemic was also seen as a “threat of an invisible danger” by contrast. Further concerns were uttered regarding the long-term consequences of the mitigation efforts, such as a deterioration of the general economic situation and future societal and psychosocial repercussions.

More adaptive responses referred to relief from performance pressure, as jobs were gone and schools were closed. They also included that patients were actively searching for ways to deal with homeschooling, particularly online school demands. Some patients actively searched for reliable information. Positive statements regarding the governmental facilities included gratefulness for living in a country with a sound health care system and social security and that “the state takes care of me (…) economically”. Regarding the psychotherapeutic setting, setting changes were being accepted, and COVID-19 was sometimes addressed as a marginal issue and sometimes discussed only at the beginning of the sessions. Patients also expressed the hope that the pandemic could be a chance to rethink society.

#### 3.1.3. Interpersonal Level

Addressed stressful environmental conditions at the interpersonal level found in all four countries regarded forced proximity at home, the possibility that loved ones might get infected, the additional burden of childcare, and homeschooling. Addressed conditions in less than four countries regarded the loss of loved ones who died from COVID-19, not being able to see friends in person, the possibility of getting infected by others, and the separation of families and couples.

The addressed maladaptive responses to these stressors included conflicts within families, fear of meeting hostile others and even domestic violence. The fact that “most patients talk about the consequences of having to be at home and having little contact with people outside the family,” as one therapist put it, at times resulted in a feeling of “unbearable closeness”. Forced proximity led to the acute re-emergence of old conflicts within partnerships and families. Disputes with neighbours were reported to have been spurred by anger or a lack of understanding towards others who would not comply with the measures. These conflicts sometimes culminated in accusations of threats of a police report. In some patients, the altered or aggressive social behaviour of others caused anxiety or the aggravation of (other) pre-existing symptoms. The fact that schools and kindergartens were closed led to feelings of fatigue and overstrain among parents and the question of where one could still find personal space not invaded by others. These problems particularly concerned parents of younger children who would now remain “isolated at home” or be “restless and difficult to deal with”. Fears and worries were addressed about the health of others, particularly of relatives, some of whom were elderly and chronically ill or living in senior citizen homes. Therapists also reported “fear of being toxic to others, literally and symbolically” or guilt of having infected others. In cases of the loss of loved ones, the reported responses were, not surprisingly, addressing feelings of grief and sorrow. The fact that partners and family members remained abroad caused longing and uncertainty.

In a more adaptive vein, patients found new ways of staying connected with others without engaging physically, a positive evaluation of having more contact with one’s family or partner and reduced interpersonal anxiety. Some patients found it relieving that “others can now relate to my experience of anxiety that I have had all my life” or that “other people do not go anywhere now either”. Others felt relieved by “not having to compare me now with my much thinner colleagues”.

#### 3.1.4. Individual Level

Patients addressed the following stressful environmental conditions at the individual level: the possibility of contracting COVID-19, having to stay at home/isolation, and a fast-changing environment.

According to the SEM, the maladaptive responses to these stressors were primarily related to fear and anxiety and aggravation or recurrence of pre-existing symptoms. Fears tended to revolve around health concerns, an uncertain future, and existential issues such as the possibility of dying and leaving the house. Among the aggravated symptoms, which we could not assign to a single cause, were depression, anxiety, addiction, panic attacks, obsessive compulsion, psychosis, and insomnia. For example, one psychotherapist stated that COVID-19 was addressed “primarily as an anxiety issue, but also as an everyday management challenge and a depression-intensifying factor due to the limited access to resources”. Re-traumatization was mentioned within the context of reactivation of early childhood trauma but also as reactivation of war trauma. A severe psychological decompensation was observed mainly in psychiatric patients. The experiences of loneliness and boredom were addressed due to having to stay at home with no access to resources or leisure time activities. Other reported consequences of staying at home were a lack of interest and feeling guilty for not doing enough. The constantly changing and uncertain situation sparked a general feeling of uncertainty, a perceived loss of control, confusion, feeling powerless, and a sense of having nothing to look forward to. Some patients also tended to express extreme views under the pressure of the situation.

Among the more adaptive responses, therapists found adaptation to the changed situation. Moreover, the pandemic was addressed as an opportunity for life changes, for example, through the revision of personal priorities, doing things that had been postponed for a long time, searching for meaning, or finding time for one’s self. The (temporary) alleviation of anxiety was addressed, and relief was due to fewer external demands. Further positive effects were the experience of calmness, improved attention, or centeredness.

### 3.2. Visualisation of Themes

**Figure 3:**
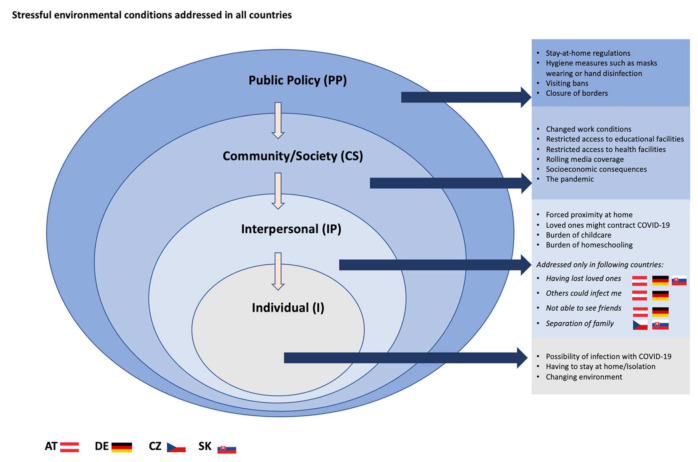
Stressful environmental conditions are addressed in all four countries at the public policy, community/society, interpersonal, and individual level.

**Figure 4:**
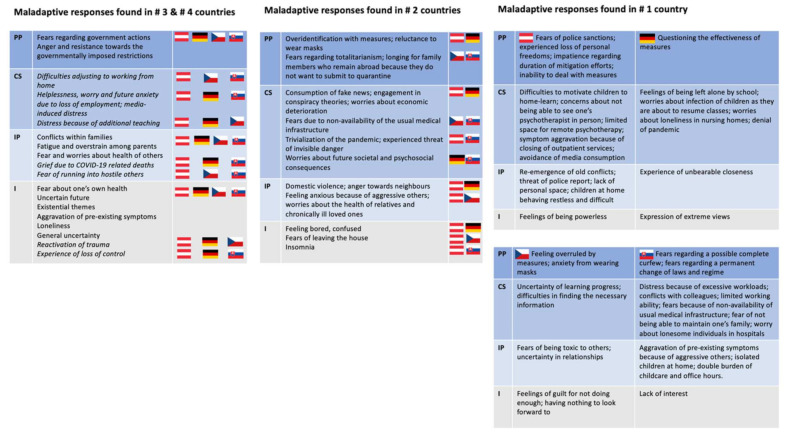
Maladaptive responses that could potentially deteriorate a patient’s health state were found in #4 and #3 countries, #2 countries, and #1 country.

**Figure 5:**
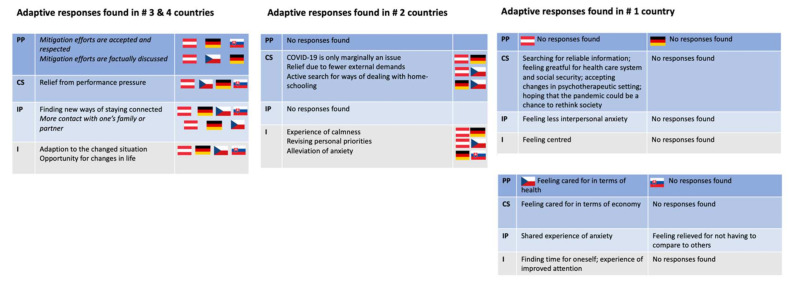
Adaptive responses that could potentially positively contribute to a patient’s health state were found in #4 and #3 countries, #2 countries, and #1 country.

Themes structured according to the SEM described narratively above are presented in abbreviated form and assigned to the countries in which they were reported in the following visualisations so that distribution patterns across countries become visible (Figure 3, Figure 4 and Figure 5).

We first show a depiction of the model of the SEM from the level of public policy to the individual level and point out which reported stressful environmental conditions refer to which level (Figure 3).

In the following, we show tables following the levels of the SEM (abbreviations for the levels are noted on the left of each table). In both figures referring to maladaptive responses (Figure 4) and adaptive responses (Figure 5), we first point out responses found in three or four countries. Then we point out responses found in two countries. Last, we address those responses found in only one country. Overall, Figure 4 and Figure 5 show the responses to the effects of stressful environmental conditions (as enlisted in Figure 3), as they trickle down from the macrosystem to the individual level (levels shown on the left), and which type of response was found in which country(ies) (countries represented by their flags as described in Figure 3).

### 3.3. The Most Frequent Word Stems Used according to Countries

The following table shows the five most frequently occurring word stems. Fear/anxiety ranks highest in all four countries. Restrictions are mentioned least frequently in Austria, and family issues are noted more often in Czechia and Slovakia than in Austria or Germany.

## 4. Discussion

Our study of immediate reactions to the pandemic referred to a timeframe from 24 March until 28 May, when the first lockdown was in place in all four participating countries. The collected responses were conceptualised following a modified version of the SEM to define public policy, community/society, interpersonal, and individual-level reactions. The resulting country-specific images were informed not only by the psychological state of the patients at the time the surveys were open but also by the social distancing guidelines and other policies and messages from the respective governments, as well as an area’s unique history. By exploring the interplay between individuals and their social-ecological context, we considered that a pandemic is disruptive at several levels, thus yielding responses at multiple levels. According to the SEM, we divided each class into three clusters: addressed stressful environmental conditions, maladaptive responses, and adaptive responses. Additionally, we pointed out the five most frequent word stems found in our data sets.

### 4.1. Stressful Environmental Conditions

The following stressful environmental conditions were addressed in all four countries: At the level of public policy, the mitigation efforts included stay-at-home regulations, closure of kindergartens and schools, visiting bans and the closure of borders. Key themes at the level of community/society were employment, restricted access to educational and health facilities, socioeconomic consequences, and the pandemic. Key themes at the interpersonal level regarded forced proximity, the possibility of infection of loved ones, childcare, and homeschooling. Key themes at the individual level were the possibility of contracting COVID-19, having to stay at home/isolation, and a changing environment. These addressed stressful environmental conditions are in line with factors brought to light by other studies on the impact of COVID-19 on individuals with pre-existing mental health conditions [13,25,31,32,33,34].

### 4.2. Maladaptive and Adaptive Responses from the Public Policy to the Individual Level

At the public policy level, maladaptive responses to the government actions to mitigate the viral spread were associated with fears in all four countries, which is not surprising, as fear/anxiety is arguably the central emotion in an individual’s response to the pandemic [23,32,51,52]. In line with the observation that the more anxious people are, the angrier and more extreme in their world views they may become [53], our results show that the governmentally imposed mitigation efforts have also caused anger and resistance among some patients in all four countries. Indeed, adherence to all rules of social distancing appears highly challenging for the vast majority of participants [54]. For the two German-speaking countries, Austria and Germany, we detected two opposing reactive tendencies: overidentification with the measures, such as being fastidious regarding hygiene rules or angry at people who transgress them, and a reluctance to follow the mitigation efforts. The observed reluctance shows as a pattern, including impatience with the duration and questioning the effectiveness of containment policies. Possibly, these dispositions were the beginnings of what later manifested itself as the movement referring to themselves as “lateral thinkers” *(Querdenker)*, which has emerged in Germany and Austria. United under the umbrella of criticism of COVID-19 policies, the movement has denied the existence of the coronavirus or at least belittled the consequences of an infection and called for protests against the government measures to reduce the spread of COVID-19 [55]. It is characterised by a profound alienation from the core institutions of liberal democracy [56]. It is now under surveillance by the German Federal Office for the Protection of the Constitution (Verfassungsschutz) (Michael Götschenberg, “Verfassungsschutz: ‘Querdenker’ werden nun bundesweit beobachtet,” Tagesschau, Available online: https://www.tagesschau.de/inland/verfassungsschutz-querdenker-103.html (accessed on 28 April 2021)). In Czechia and Slovakia, early reactions towards COVID-19 policies appeared more driven by fear. Czech and Slovak patients’ association of the mitigation efforts with totalitarianism might be best explained by their history of communist rule until 1989 and with memories thereof, which the mitigation efforts have brought to the surface. Slovak patients also expressed fears regarding a complete curfew and a permanent change of rules and regime. Indeed, one of the most common hoaxes in Slovakia was the report that soldiers were moving into the capital Bratislava and that martial law was being imposed because of the spread of the coronavirus [57]. In Czechia, misinformation was spread that the virus was created as a biological weapon [58]. Another finding specific to Czechia and Slovakia is longing for family members who remained abroad because they did not want to submit to quarantine regulations. We think that these reactions reflect a consequence of transnational work arrangements, as workers from Eastern countries often travel to Austria for work. For example, Austria’s long-term care services system relies heavily on live-in migrant carers (personal carers) from the neighbouring countries. The measures introduced to limit the spread of COVID-19 across Europe in spring 2020 included the temporary closure of borders. For live-in carers and other workers from neighbouring countries, such as Czechia and Slovakia, they could no longer commute between the Austrian households they work in and their countries of origin. A study showed that during the initial stage of the pandemic, workers’ interests were subordinated to the interests of care recipients and agencies [59], and we suspect that this shows in our data. Among the responses found in Austria are fears of police sanctions and the experience of loss of personal freedom. These fears and experiences were inspired by Austria’s quick and harsh measures involving frequent police patrolling and penalties for transgressions of public policies, particularly in larger cities, (Available online: https://www.derstandard.at/story/2000116206367/die-polizei-und-das-virus-vernaderung-verwarnung-und-der-neffentrick, accessed on 28 April 2021) as the Austrian government reacted under the impression of the events in neighbouring Northern Italy, where excess mortality was observed during the first wave of the outbreak [60]. Additionally, we conducted this survey earlier in Austria than in the other three countries. When it was open in Austria, there was no end in sight to the measures, which also might have contributed to the observed impatience regarding the duration of the mitigation efforts in Austria. Adaptive responses at the public policy level indicate that the mitigation efforts were factually discussed and accepted. Overall, the adaptive responses were less multifaceted than the maladaptive ones.

At the combined community and societal level, the resistance pattern identified among German-speaking patients at the public policy level is paralleled by a pattern of consumption of fake news and conspiracy theories. This is in line with a UK study on health-protective behaviour that found a negative relationship between COVID-19 conspiracy beliefs and COVID-19 health-protective behaviours [61], which gives us a hint as to how the two phenomena might be connected. Other maladaptive responses identified at the community and societal level do not appear typical for one country or region but seem to spread across nations. The reported worries, future anxieties, and feelings of helplessness were directed at the possibility of job loss and difficulties adjusting to working from home. Being among the most critical factors, loss of work [62] and adverse work conditions [63] contributed to a deterioration of mental health throughout the pandemic worldwide. Distress was also reported to result from having to school one’s children at home, uncertainty regarding the progress of learning, worry about the quality of education, feeling left alone by schools, and difficulties in motivating children to learn. This is in line with findings from a study about homeschooling in seven European countries, which indicate that homeschooling had adverse effects on both parents and children [64].

Moreover, our findings from Germany, Slovakia, and Austria suggest that increased exposure to media fueled people’s cycles of distress, as was presented by the literature [65,66,67]. What we framed as adaptive responses for Austria and Germany, namely that “the pandemic is barely an issue”, in the vein of the above-described pattern of resistance could also be interpreted as a “trivialisation of the pandemic” rather than being in a good mood. Fears regarding the non-availability of the usual medical infrastructure were only reported for Czechia and Slovakia. In Czechia, the concern of the non-availability of medical infrastructure was one of the most-rated sources of fear at the onset of the pandemic [68]. On the other hand, despite considerable distrust towards the public health system among German-speaking patients, the fear of the unavailability of health care was not mentioned even once. This fear is less likely to be found in nations with a high income, such as Austria and Germany.

Nevertheless, reduced and inadequate access to mental health support services, such as the closure of outpatient services, was reported in Austria. According to the literature, such shortfalls in mental health care are among the most common factors that have negatively impacted individuals with pre-existing mental health conditions [25,32,34]. The Austrian therapists who participated in this study, too, confirmed that these factors had caused concern and symptom aggravation among their patients. Moreover, studies conducted with the same therapists have demonstrated that psychotherapeutic care in Austria was less available and more often remote than in the other surveyed countries within the survey period [42,43]. Common adaptive responses at the level of community/society referred to relief from performance pressure and additional (not further specified) external demands. We think that these responses are only adaptive in the sense of the SEM as they contribute, at least in the short term, to more serenity in a challenging situation. However, since individuals draw satisfaction from their social withdrawal only a short while after escaping real-world stresses, and since longer-lasting social isolation is known to increase loneliness gradually, such responses are a crucial risk factor for mental health deterioration in the long term [69].

The pandemic was addressed in conjunction with interpersonal hostility and social conflict in all four countries at the interpersonal level. Increases in enmity and social strife are unsurprising, as pandemics have historically exacerbated community tensions or created new ones [16]. A UK study has shown that more than half of (*n* = 1255) study participants reported having had arguments, felt angry, or fallen out with others because of COVID-19. Since tension may be influenced by disagreements about the levels of risk posed by COVID-19 or how to adhere to containment efforts, anger and conflict could be associated with factors such as lack of knowledge or endorsement of conspiracy theories [61]. Although an increase in domestic violence shortly after announcing the stay-at-home regulations was reported in many regions [70], in our study, an explicit mention of domestic violence was only found for Austria and Germany. This finding does not necessarily indicate no domestic violence in Czechia and Slovakia. It could also mean that domestic violence is less likely to be addressed in former communist bloc countries [71]. In fact, in Slovakia, hospitals were advised by the government not to refuse to treat victims of domestic and sexual violence even during the lockdown period when non-essential visits to medical facilities were discouraged [57]. Other responses found in all four countries refer to fatigue and overstrain among parents who cared for their children as schools and kindergartens were closed. Studies confirm that families with minors have been hit particularly hard by COVID-19 as parents are left alone with homeschooling and childcare [72,73], with feelings of social isolation and loneliness [74]. Moreover, parental adverse childhood experiences are likely found in mental health patients. They are an essential determinant, as they increase vulnerability to stress and thus potentially harmful parenting behaviour during the pandemic [73]. Since parents’ psychological distress potentially sets a vicious circle of parental and child emotional dysregulation [75], we conclude that parenthood during the pandemic must have been a massive additional stress factor for individuals with pre-existing mental health conditions. Worries about the health of others and grief due to COVID-19 related deaths are themes not only found in our data but also in the literature, where mainly older adults appear to be concerned [76,77]. We would like to point out that grieving and worrying about others are expected human reactions. They are maladaptive only in the sense of the SEM, as they could lead to the attendance of collective mourning rituals and thus foster infection. Official traditional mourning rituals were impossible because of stay-at-home regulations [78], which may have contributed to an even more stressful experience. Since death themes were mentioned frequently, we assume that psychotherapy has played an essential role in processing grief for the deceased. Among the adaptive responses, finding new ways of staying connected was practised in all four countries. Furthermore, having more contact with one’s partner or family was not only experienced as a burden but also evaluated positively. The literature shows that activating social networks, albeit remotely, is essential to counteract isolation [8,79,80] and has been observed as a protective factor during the initial stage of the pandemic also among individuals with pre-existing health conditions [33,34]. According to our study, some people also felt relief from feeling that others, too, experienced anxiety or from not having to face interpersonal situations anymore. Such reactions were also found in another cross-country study about the early impact of the pandemic on people with mental health conditions [32]. 

At the individual level, the following common maladaptive responses were found in all four countries: fear and anxiety about one’s health, uncertainty about the future, themes of one’s death and existence, loneliness, general uncertainty, and an aggravation of symptoms of mental illness. Fears related to contracting COVID-19, symptom aggravation, loneliness and “uncertainty about the future” have been found among individuals with mental illness [13,31,32,33] but also among the general population [81,82]. Loneliness as a consequence of self-isolation during the pandemic has been defined as a signature mental health concern in the era of COVID-19 [83] and found to be significantly positively correlated with anxiety, depression, and stress levels [84]. We want to point out that these emotional reactions are not necessarily maladaptive psychologically. However, according to the SEM, they may prevent optimal adaptation to the challenges of an epidemic situation.

Among the adaptive responses, we found that patients in all four countries had adapted to the new situation and that some could frame it as an opportunity for life changes. Other, less common reactions comprised the experience of calmness, revision of personal priorities, and anxiety alleviation. These abilities to mobilise skills and resilience and the experience of fewer symptoms during the pandemic have been observed in other countries as well [32]. From a psychological view, what we framed as adaptive reactions could mean that the patients’ pathology and the requirements of the pandemic situation just happened to be well-aligned. For example, individuals with depression and anxiety experienced lower pressure for social interaction [21]. However, according to the SEM, these reactions were adaptive in the given situation, fostering calmness and serenity.

### 4.3. Word Stem Analysis

As can be seen from our word stem analysis, reactions related to “fears”, “anxieties”, and “worries” are among the most frequently mentioned, followed by “restrictions” in the two German-speaking countries and by “family-” themes in Czechia and Slovakia, only then followed by “restrictions”. As discussed above, the more frequent mentioning of family themes in Czechia and Slovakia compared to Austria and Germany may point to the theme of longing for family members who remained abroad. It could also mean that in the former communist bloc countries, there is still a more prominent accent on the value of the family, given by history when the family was perceived as the bedrock of the Marxist Leninist political ideology [85].

Overall, results from word stem analyses indicate that the adverse reactions predominated.

### 4.4. Limitations

This study has several limitations. One limitation is its cross-sectional design, which did not allow for evaluating the impact of post-COVID conditions on the mental health of patients of psychotherapists. We want to emphasise that we did not use standardised scales or controls since the design of this study is purely descriptive.

Our study participants had to write their responses to a written question rather than being interviewed face-to-face, which limited our ability to derive more coherent and contextually embedded information. Since mental health care patients are a vulnerable group, we did not approach them directly during the pandemic. Instead, we based our approach on the method of participative observation conducted by mental health professionals. Clinicians provided us with data on how they witnessed and interpreted the effect of the pandemic on their patients, which further eliminated heterogeneity in the expression of individual experience. However, it should be kept in mind that a research team might not get the same reports as psychotherapists on psychotherapy patients’ experiences of the COVID-19 pandemic. As patients and psychotherapists usually have a strong bond, patients might disclose more information to their psychotherapists than to researchers.

Our study drew on different sample sizes, which partly has to do with different situations in the field of psychotherapy in the four surveyed countries. The largest sample of psychotherapists (A) relates to the highest availability of psychotherapeutic care in the country. Statistically, there are more than 10.500 psychotherapists in Austria (one psychotherapist for 833 citizens). In contrast, there are only 472 psychotherapists in Czechia (one psychotherapist for 22.669 citizens), similarly in Slovakia (one psychotherapist available for 15.227 citizens). The Austrian sample was significantly larger than the others (A: 1547, CZ: 112, DE: 130, SK: 96), allowing for greater response variability, which may have biased the results. However, we would like to point out that in qualitative descriptive research, small sample sizes are common [40]. The sample sizes, including around 100 participants, are already substantial for qualitative research.

As this study did not comprise a control sample consisting of the general population, we cannot say whether the impact of the COVID-19 situation on psychotherapy patients is the same or different compared to the general population

It should also be noted that, whereas the survey period in Austria was open during the initial phase of the first lockdown, it was open seven to eight weeks after lockdown measures were initiated and after restrictions began to be lifted in the other three countries. At the measurement time, Austria had the highest number of official cases (Figure 1). Slovakia scored highest, and Czechia scored lowest in terms of strictness of measures (Figure 2). It should be considered that, since the survey took place earlier in Austria than in the other three countries, no measures were lifted yet there at the time of the study. Furthermore, it was still unclear when measures would be lifted, which presumably entailed an atmosphere even more charged with insecurity and fears. These differences, too, may have biased the results.

It is crucial to remember that the results of our study represent a snapshot of a specific timeframe and that we captured the situation in Austria at an earlier point in time than in the other three countries. The longer the lockdown, the more health problems might arise. Therefore, the lockdown impacts may have changed dynamically depending on the duration of the lockdown and the degree of the restrictions.

## 5. Conclusions

Being prepared for new pandemic events is essential for clinicians, as we have seen an increase in global fears of disease and infectious agents [86]. New pandemics are likely to emerge as people are exceedingly mobile and likely to live in densely populated cities [87]. This study thus explored stressful environmental conditions and responses from the public policy level to the individual level in four European countries among patients with pre-existing mental health conditions. A qualitative content analysis revealed that reactions that could potentially deteriorate a patient’s health state were more multifaceted than responses identified as adaptive. Two country-specific observations emerged: A pattern of resistance, including reluctance to mask-wearing, questioning the effectiveness of policies, and being prone to fake news and conspiracy theories was found in the two German-speaking countries. In Czechia and Slovakia, on the other hand, we identified a pattern of fear regarding totalitarianism and a pattern of longing for family members who remained abroad due to transnational work arrangements that were affected by the quarantine regulations. An analysis of the most frequent word stems out of the responses from each country shows that fears, anxieties, and worries were the most commonly addressed themes, followed by themes regarding restrictions and family.

This study provides a holistic view of mental health patients’ concerns by documenting the influence of socio-environmental factors on their experience at the onset of the pandemic in Europe. It allows a view beyond the psychological interpretation of reactions by showing that the alignment of pathologies with the demands of the environment can also be favourable in a pandemic situation. The results have implications for clinical practice and public policy in times of a pandemic, as they can help trace the influences of socio-environmental factors on individual experience.

## Figures and Tables

**Table 1:** The percentage of the five most frequent word stems in all four countries was ordered by total. * Fear/anxiety refers to only one word stem in the German language but to two word stems in the Czech and Slovak languages. More comprehensive tables by country with word stems occurring with more than 5% frequency in the responses are included in the supplementary material.

	%			
English Equivalent to Local Word Stem	Austria	Germany	Czechia	Slovakia
Fear/anxiety (anxieties, anxious…) *	28.5	41.7	24.0 24.0	24.7 23.6
**worry, worries**	20.5	19.2	36.5	20.2
**restriction(s), limitation(s), -ing**	12.7	25.0	18.3	16.9
**uncertainty, -ies**	8.9	6.7	20.2	16.9
**family, -ar, -ies, inkl. parent(s)**	5.8	6.7	19.2	19.1

## Data Availability

Data are available upon reasonable request.

## Supplementary Materials

The following supporting information can be downloaded at: https://www.mdpi.com/article/10.3390/ijerph19116825/s1; supplement to Table 1: Comprehensive tables by country with word stems with a frequency of more than 5% in the responses.
