# Supplementary material for: Psychotherapists’ Reports regarding the Impact of the COVID-19 Pandemic on Their Patients: A Cross-National Descriptive Study Based on the Social-Ecological Model (SEM)
Source: Int J Environ Res Public Health. 2022 Jun 2;19(11):6825. doi: 10.3390/ijerph19116825 (PMC9180390; doi:10.3390/ijerph19116825)
Supplement: Supplementary file 1 [file ijerph-19-06825-s001.zip › ijerph-1731946-supplementary.pdf]

Comprehensive tables by country with word stems with more than 5% frequency in the responses. In total, 1885 psychotherapists participated. Out of this, 1690 gave answers to our question.

Austria (A)

Table S1 Word stems in psychotherapists' (n=1377) articulations of how COVID-19 was addressed in psychotherapy sessions with a frequency of more than 5% in Austria.

German word stem	***n***	***%***	***English equivalent***
angst / ängst	392	28.5	fear, anxiety (anxieties, anxious…)
sorge	282	20.5	worry, worries
schränk	175	12.7	restriction(s), limitation(s), -ing
(un-)sicher	123	8.9	(un-)safe, (un-)secure, (un-)certain
leben	122	8.9	life, living
änder	109	7.9	change(s)
über	101	7.3	about, over
situation	100	7.3	situation
tag	100	7.3	day
isol	96	7.0	isolation
belast	93	6.8	burden(ed), strain(ed)
zukunft	86	6.2	future
auswirk	84	6.1	effect
exist	81	5.9	existence, exisiting
famili	80	5.8	family, -ar, -ies
alltag	77	5.6	daily routine, everyday life
einsam	73	5.3	lonely, solitary
perso / ö	70	5.1	person, personal

Germany (DE)

Table S2 Word stems in psychotherapists' (n=120) articulations of how COVID-19 was addressed in psychotherapy sessions with a frequency of more than 5% in Germany.

German word stem	***n***	***%***	***English equivalent***
angst / ängst	50	41.7	fear, anxiety (anxieties, anxious…)
schränk	30	25.0	restriction(s), limitation(s), -ing
sorge	23	19.2	worry, worries
schul / school	23	19.2	school
belast	20	16.7	burden(ed), strain(ed)
kontakt	12	10.0	contact
soziale	11	9.2	social
änder	10	8.3	change(s)
fehlende	9	7.5	missing, absent
über	9	7.5	about, over

alltag	8	6.7	daily routine, everyday life
stress	8	6.7	stress
famili	8	6.7	family, -ar, -ies
unsicher	8	6.7	uncertain
leben	7	5.8	life, living
frage	7	5.8	question, questioning
kind	7	5.8	child(ren)

Czechia (CZ)

Table S3 Word stems in psychotherapists' (n=104) articulations of how COVID-19 was addressed in psychotherapy sessions with a frequency of more than 5% in Czechia.

Czech word stem	***n***	***%***	***English equivalent***
obav	38	36.5	worry, worries
strach	25	24.0	fear
úzkost	25	24.0	anxiety (anxieties, anxious…)
nejisto	21	20.2	uncertainty, -ies
rodi	20	19.2	family, -ar, -ies, inkl. parent(s)
omez	19	18.3	restriction(s), limitation(s), -ing
život	17	16.3	life
blízk	14	13.5	close, -ness
změn	14	13.5	change(s)
prac+prác	13	12.5	work, job
izolac	12	11.5	isolation
opatření	10	9.6	measure(s)
tíž	10	9.6	difficulty, -ies, trouble(s)
zdrav	10	9.6	health
budoucnost	9	8.7	future
dět	9	8.7	children
finan	9	8.7	finance, -ial
karant	8	7.7	quarantine
situac	8	7.7	situation, -al
ztrát	8	7.7	loss
dopad	7	6.7	impact
možnost	7	6.7	(im)possibility, only once possitive
partner	7	6.7	partner
téma	7	6.7	theme, topic
vztah	7	6.7	relationship
dom	6	5.8	home, domestic, house(hold)
lid	6	5.8	human(s), people
nov	6	5.8	new
osob	6	5.8	person(al)
poci	6	5.8	feel

Slovakia

Table S4. Word stems in psychotherapists' (n=89) articulations of how COVID-19 was addressed in sessions of psychotherapy with a frequency of more than 5% in Slovakia.

Slovak word stem	***n***	***%***	***English equivalent***
strach	22	24.7	fear
úzkos	21	23.6	anxiety (anxieties, anxious…)
obav	18	20.2	worry, worries
rodi	17	19.1	family, -ar, -ies, inkl. parent(s)
neistot	15	16.9	uncertainty, -ies
obmedz	15	16.9	restriction(s), limitation(s), -ing
izol	12	13.5	isolation
život	12	13.5	life
prác	12	13.5	work, job
strat	11	12.4	loss
zmen	11	12.4	change(s)
soc	11	12.4	social
tém	10	11.2	theme, topic
kontakt	10	11.2	contact
det	8	9.0	child(ren)
zdrav	7	7.9	health
blízk	7	7.9	close, -ness
finan	7	7.9	finance, -ial
chor	6	6.7	ill / sick -ness
karant	6	6.7	quarantine
probl	6	6.7	problem -atic
dom	6	6.7	home, domestic, house(hold)
ťaž	6	6.7	difficulty, -ies, trouble(s)
starost	6	6.7	concern(s)
opatren	5	5.6	measure(s)
možnos	5	5.6	(im)possibility
situac	5	5.6	situation
spojen	5	5.6	connected
vzťah	5	5.6	relationship
